# Supplementary material for: The discovery of novel noncoding RNAs in 50 bacterial genomes
Source: Nucleic Acids Res. 2024 Apr 22;52(9):5152–65. doi: 10.1093/nar/gkae248 (PMC11109978; doi:10.1093/nar/gkae248)
Supplement: gkae248_Supplemental_Files [file gkae248_supplemental_files.zip › Narunsky 50 Genomes RNA Motifs Files.zip!/Publication/54-P_aestuarii/Figures/LRC-54-29-NC_011059_1_IGR_76_348694_349133.sto.pdf]

LRC-54-29-NC_011059_1_IGR_76_348694_349133.sto.r2r

5´-○○○●CUGYUGCG●●Y●●CGAU●GC●RCGUUGR●YGG●GYYCGRA●UCCUCACGURCU●UAU●●ACG●●YC●G●U●CU●YRYUCCG●YYGCCUUGC●CUC●●R
